# Little strokes, big trouble and more…

**DOI:** 10.4103/0972-2327.44552

**Published:** 2008

**Authors:** Sanjeev V. Thomas

**Affiliations:** Professor of Neurology and Editor, Annals of Indian Academy of Neurology, Department of Neurology, SCTIMST, Trivandrum, India. E-mail: editor@annalsofian.org

On 26 November 2008, the people of India experienced again the gory of terrorism. This time it was a series of brutal attacks on civilian locations in Mumbai that took the lives of more than 180 innocent persons. Our forces had displayed extraordinary valor, tact and tenacity while tackling the killers and did not hesitate to give up their lives to uphold our security. We record our deepest condolence to all the bereaved family members and salute the martyrs who saved our lives. Terrorism is a growing global menace. It does not achieve any political gain to anybody, but destroys the peace and comfort of many. Each nation has to spend more money and energy to ward off such threats. It is important that each one of us resolve to fight against such evils and exert our influences in the community to turn the opinion against such movements.

Worldwide, more than 5.7 million people die of stroke every year. More than 80% of stroke occurs in underdeveloped countries that have only meager facilities to cope with the situation. According to observations from the Framingham study, people with high blood pressure (≥ 140/90 mm Hg) had twice the lifetime risk of stroke when compared to those with normal blood pressure (≤ 120/80 mm Hg).[1] WHO, World Federation of Neurology, and World Stroke Organization have started the global stroke initiative for stroke surveillance, public education on stroke risk factors, and to inform healthcare providers on current stroke care options. As part of this initiative, 29^th^ October is observed as World Stroke Day. The theme of World Stroke Day 2008 is ‘Little Strokes, Big Trouble.’[2] Early changes in physiological variables are important determinants of mortality and late morbidity in persons with acute stroke. These variables deserve careful management in the early phase of stroke, yet there are few reliable guidelines to assist the physician. In this issue, we have included an extensively researched review article on the physiological variables of stroke. Professor Graham Venables, in his oration at the Annual Meeting of the Indian Academy of Neurology reiterated the importance of translating research in to clinical practice with reference to thrombolytic therapy. We have reproduced the text of his oration in this issue of the journal.

The daunting task that we now face in India is how to overcome the organizational difficulties and make therapy available to patients with stroke within the window period. In this context, the paper that describes the experience at an institution in Uttar Pradesh is an eye opener. The authors have demonstrated that despite all the practical difficulties prevailing in India, it *is* possible to deliver hyperacute thrombolysis to deserving patients living in the semiurban and rural areas of India.

The ultimate goal of all efforts of a nation at the macro and micro level is to improve the quality of life of its citizens. Unfortunately, the medical fraternity has so far been largely preoccupied with pathology and morbidity and failed to give sufficient importance to health-related quality of life issues when evaluating new therapeutic modalities. Recently, this has been recognized, and most of the prospective studies now also address the question of how a treatment modality would influence the quality of life of the end-user and the caregivers. There are several generic and specific instruments to evaluate the quality of life of persons with various neurological disorders. Quality of life should be evaluated from the perspective of the patients and their caregivers, rather than from that of the medical professionals. In that sense, the economic burden due to the disease and its treatment, and several other relatively intangible components such pain, stress, and social disruption, would have to be addressed while evaluating quality of life. In this issue of the journal we have two papers that deal with the quality of life of persons with neurological disorders. We hope that these papers will stimulate our readers to pay attention to this aspect in their practice.

The present editorial board and advisory committee have been behind this journal for the past 3 years. The positive feedback that we have received from the honorable members of the academy and other readers indicates that the journal has been doing well these past few years. Several persons have devoted much time and energy to raise the standards of the journal. The office bearers and several distinguished members of the Academy have extended their wholehearted support to us. The direct grant from the Academy and generous donations from the organizers of the annual meetings, particularly of the 15^th^ annual meeting in Mumbai, has improved the fragile financial situation of the journal. We wish to place on record our appreciation to the publishers, Medknow, for maintaining the high technical standards of this journal. We also wish all our readers a Happy New Year.
